# Optimal Sequential Strategies for Antibody-Drug Conjugate in Metastatic Breast Cancer: Evaluating Efficacy and Cross-Resistance

**DOI:** 10.1093/oncolo/oyae055

**Published:** 2024-04-04

**Authors:** Meiting Chen, Riqing Huang, Rishang Chen, Fei Pan, Xiujiao Shen, Haifeng Li, Qixiang Rong, Xin An, Cong Xue, Yanxia Shi

**Affiliations:** Department of Medical Oncology, State Key Laboratory of Oncology in South China, Guangdong Provincial Clinical Research Center for Cancer, Sun Yat-sen University Cancer Center, Guangzhou, People’s Republic of China; Department of Medical Oncology, State Key Laboratory of Oncology in South China, Guangdong Provincial Clinical Research Center for Cancer, Sun Yat-sen University Cancer Center, Guangzhou, People’s Republic of China; Medical Oncology Department III, Central Hospital of Guangdong Nongken, Zhanjiang, People’s Republic of China; Department of Breast Medical Oncology, Shandong Cancer Hospital and Institute, Shandong First Medical University and Shandong Academy of Medical Sciences, Jinan, People’s Republic of China; Department of Pathology, State Key Laboratory of Oncology in South China, Guangdong Provincial Clinical Research Center for Cancer, Sun Yat-sen University Cancer Center, Guangzhou, People’s Republic of China; Department of Medical Oncology, State Key Laboratory of Oncology in South China, Guangdong Provincial Clinical Research Center for Cancer, Sun Yat-sen University Cancer Center, Guangzhou, People’s Republic of China; Department of Medical Oncology, State Key Laboratory of Oncology in South China, Guangdong Provincial Clinical Research Center for Cancer, Sun Yat-sen University Cancer Center, Guangzhou, People’s Republic of China; Department of Medical Oncology, State Key Laboratory of Oncology in South China, Guangdong Provincial Clinical Research Center for Cancer, Sun Yat-sen University Cancer Center, Guangzhou, People’s Republic of China; Department of Medical Oncology, State Key Laboratory of Oncology in South China, Guangdong Provincial Clinical Research Center for Cancer, Sun Yat-sen University Cancer Center, Guangzhou, People’s Republic of China; Department of Medical Oncology, State Key Laboratory of Oncology in South China, Guangdong Provincial Clinical Research Center for Cancer, Sun Yat-sen University Cancer Center, Guangzhou, People’s Republic of China

**Keywords:** metastatic breast cancer, antibody-drug conjugate, trastuzumab emtansine, trastuzumab deruxtecan, disitamab vedotin, sacituzumab govitecan

## Abstract

**Background:**

The optimal sequential strategy for antibody-drug conjugates (ADCs) in breast cancer remains uncertain. This study aimed to evaluate the efficacy and potential resistance of second ADC (ADC2) following the first ADC (ADC1) in human epidermal growth factor receptor 2 (HER2)-positive and HER2-low MBC.

**Methods:**

This retrospective, multicenter, real-world study enrolled patients with MBC who received at least 2 different types of ADCs in 3 hospitals in China between July 1, 2017 and May 1, 2023. Outcomes included the objective response rate (ORR) for ADC1 and ADC2, progression free survival 2 (PFS_2_), defined as the time from initiation of ADC2 to progression, and overall survival (OS).

**Results:**

Seventy-nine female patients were included, 64 of whom had HER2-positive disease. The ORR for ADC2 with similar payload of ADC1 was found to be 5.3%. When switching to a different payload, the ORR of ADC2 increased to 22.6%. The PFS_2_ for ADC2 remained similar regardless of whether the payload was similar or different. Switching to different payload showed a higher ORR in patients with rapid progression and a durable response longer than 6 months (41.2% vs 15.0%). Specifically, significantly longer PFS_2_ and OS were seen in patients treated with trastuzumab deruxtecan (T-Dxd) compared to those treated with disitamab vedotin (RC48) after progression from trastuzumab emtansine (T-DM1; median PFS_2_ 5.37 months vs 3.30 months, HR = 0.40, 95% CI 0.17-0.93, *P* = .034; median OS 50.6 months vs 20.2 months, HR = 0.27, 95% CI 0.08-0.91, *P* = .034). For patients who progressed after T-Dxd, the median PFS_2_ was 6.05 months for those treated with RC48 versus 0.93 months for those treated with T-DM1 (HR = 0.03, 95% CI 0.002-0.353, *P* = .0093). Genomic analysis revealed that alternation of retinoblastoma1 was significantly associated with superior PFS.

**Conclusion:**

The alternation of payload achieves different responses in different settings. T-Dxd followed by RC48 may be a potentially beneficial strategy in HER2-positive disease. Further research is needed to elucidate the mechanism of cross-resistance.

## Teaser

This study explored the optimal sequencing for antibody-drug conjugates (ADCs) by analyzing the different strategies using at least 2 types of ADC for metastatic breast cancer with real-world data.

**Implications for Practice**

This study focused on the resistance and efficacy of second antibody-drug conjugate (ADC) after first ADC for HER2-positive and HER2-low metastatic breast cancer. Trastuzumab emtansine (T-DM1) followed by trastuzumab deruxtecan (T-Dxd) showed superior progression-free survival and overall survival than T-DM1 followed by disitamab vedotin (RC48). T-Dxd followed by RC48 may be one potentially beneficial treatment in HER2-positive disease compared with T-Dxd followed by T-DM1.

## Introduction

In recent years, several novel antibody-drug conjugates (ADCs) have been approved for the treatment of breast cancer, particularly human epidermal growth factor receptor 2 (HER2) positive metastatic breast cancer (MBC).^1^ Trastuzumab emtansine (T-DM1) was the first ADC to be approved in solid tumors, demonstrating a significant survival benefit in the EMILIA trial.^2^ At that time, T-DM1 was recommended as second-line treatment for HER2-positive MBC. Fam-trastuzumab–deruxtecan (T-DXd) demonstrated an impressive benefit over lapatinib and capecitabine (LX), or trastuzumab and capecitabine(HX) after failure of T-DM1.^3^ However, a limitation for DESTINY-Breast 02 trial was the restriction to the treatment of physician’s choice arm of capecitabine plus either trastuzumab or lapatinib, as many other novel HER2-directed drugs have been approved in the late lines in recent years, such as trastuzumab duocarmazine, with significant activity in patients pretreated with T-DM1.^4^ The proper sequencing for T-DM1 followed by T-Dxd or T-DM1 followed by another HER2 ADC was still unknown. In the DESTINY-Breast 03 study, T-Dxd presented compelling efficacy compared with T-DM1, and T-Dxd has been recommended as second-line therapy for patients with HER2-positive MBC since 2022.^5^ The significant superior survival advantage might lie in the biochemical difference in payload and linker between T-Dxd and T-DM1, and the induction of a bystander-killing effect.^6^ In metastatic triple-negative breast cancer and endocrine-resistant hormone positive, HER2 negative disease, sacituzumab govitecan (SG), an ADC targeting trophoblast cell-surface antigen 2 (TROP-2) with an SN38 payload, presented survival benefit compared with second-line chemotherapy.^7,8^ In HER2-low expression MBC, defined as 1+ on immunohistochemical (IHC) analysis, or 2+ and negative results on in situ hybridization, T-Dxd showed longer PFS and overall survival (OS) than physician’s choice of chemotherapy.^9^ Disitamab vedotin (RC48), an innovative HER2-targeting ADC with a cleavable linker and a potent microtubule inhibitor payload MMAE that has a bystanding effect in tumor cell killing, showed promising antitumor activity in heavily treated HER2-positive and HER2-low disease.^10^ However, the real-world efficacy and the cross-resistance for RC48 in HER2-positive and HER2-low disease remained uncertain.

With the rapidly expanding availability of novel ADCs targeting different antigens and payloads, it immensely expands our ability to treat a wide range of subtypes of MBC. However, challenges for optimal sequencing of different ADCs still exist and the cost-effectiveness of different ADCs raises our concern.^11^ T-Dxd showed superior benefit to HX and LX after progression from T-DM1, but the comparison between T-Dxd and another HER2 ADC after T-DM1 was unknown. In SABCS 2023, several studies included HER2-low MBC and mainly focused on the sequential use of SG and T-Dxd, but showed different results on the PFS benefit. It was indicated that more evidence on drug resistance of ADCs was needed. In addition, the strategy for RC48 as an alternative for HER2-low disease was not reported in the above study.^12-14^ The comparison of the strategy of sequencing different ADCs with similar payloads and different payloads is lacking. Sequential use of different conjugates targeting the same antigen might be effective, but the evidence remained insufficient. Therefore, our study attempted to explore the optimal sequencing for ADCs by analyzing the different strategies using at least 2 types of ADCs for MBC with real-world data.

## Materials and Methods

### Study Design and Patients

This retrospective, multicenter, real-world study included patients with locally advanced or MBC who received more than one type of ADC at the Sun Yat-Sen University Cancer Centre (SYSUCC), Guangdong Nongken Central Hospital, and Shandong Cancer Hospital and Institute between July 1, 2017 and May 1, 2023. The inclusion criteria were (1) histologically confirmed breast cancer, unresectable or metastatic disease at enrollment, (2) received at least 2 types of ADCs including T-DM1, T-Dxd, RC48, and SG, (3) available response assessments, (4) locally advanced or metastatic cancer, and (5) complete clinical profiles. The study protocol was approved by the ethical committee of the Sun Yat-Sen University Cancer Centre. The requirement for individual informed consent was waived by the committee due to the retrospective nature of the study.

### Data Collection and Definitions

Data were extracted from the medical records and included the patient demographics, tumor characteristics, treatment, standard laboratory tests, and image scans. Generally, the patients were treated with each type of ADC until disease progression, intolerable toxicity, or death based on the physicians’ experience. ADCs were generally administered according to their instructions. According to the antibody and payloads of ADC1 and ADC2, we classified all patients into 4 groups, group 1 (G1) for similar antibody and similar payload, group 2 (G2) for similar antibody and different payload, group 3 (G3) for different antibody and similar payload, and group 4 (G4) for different antibody and different payload.

HER2 expression was mainly detected by immunohistochemistry (IHC). The IHC scores were assessed according to the HER2 testing guidelines for breast cancer.^15-17^ The HER2 gene amplification could also be evaluated by fluorescence in situ hybridization (FISH), according to the HER2 test guidelines for breast cancer.^15-17^ Her2-low status was defined as IHC 1+ or IHC 2+ and FISH negative. Hormone receptor-positive disease was defined as immunoreactive for estrogen or progesterone receptor in ≥1% of tumor cell nuclei according to local testing.

The objective response was defined as a response sustained for a minimum of 2 consecutive imaging evaluations at least 4 weeks apart. The disease was evaluated using RECIST version 1.1 for response assessment. CT was performed routinely at baseline and every 6 weeks. Follow-up CT scan data were collected for 2 years or until progressive disease (PD).

Survival was measured from initiation of therapy until the event of interest (death or progression). Disease control rate (DCR), ORR, PFS, and OS were analyzed. PFS_1_ and PFS_2_ were measured from the time from treatment initiation of first ADC, and second ADC until disease progression, or death, respectively. PFS was defined as the sum of PFS_1_ and PFS_2_. The DCR was calculated as the proportion of patients achieving a complete response (CR), partial response (PR), or stable disease (SD). The ORR was calculated as the proportion of patients achieving a CR or a PR. The duration of response (DOR) is defined as the time from the first evaluation of CR, PR, or SD to PD. Follow-up was censored on August 24, 2023.

Genomic testing encompassed hybrid capture-based targeted next-generation sequencing (NGS) on the Illumina sequencing platform to identify targetable alterations at the molecular diagnostics department of SYSUCC. Initially, DNA was extracted and sheared from archival formalin-fixed paraffin-embedded tumor along with matched normal tissues. Subsequently, sequencing libraries were generated, ensuring a consistent median depth (>500×), and assessed for somatic variants, including single nucleotide variants, small insertions, and deletions, copy number alterations, and gene fusions/rearrangements. To calculate tumor mutation burden, the number of somatic, coding, nonsynonymous single nucleotide variants, and insertions and deletions mutations per megabase of the genome examined was defined.

### Statistical Analysis

SPSS 25.0 (IBM, Armonk, NY, USA), Prism 5.01 (GraphPad Software Inc., San Diego, CA, USA), and R 4.0.2 (The R Project for Statistical Computing, www.r-project.org) were used for statistical analysis. The study population for all analyses included the patients enrolled in the study who had received at least 2 types of ADCs. Descriptive statistics were used to summarize patient characteristics, treatment administration, and antitumor activity. Hypothesis testing for categorical variables was performed using the chi-squared test or Fisher's exact test. OS and PFS were analyzed using the Kaplan-Meier method and Cox proportional hazard models. Median follow-up time was analyzed using reverse Kaplan-Meier method. Two-sided *P* < .05 were considered statistically significant.

## Results

A total of 458 patients with MBC treated with at least one type of ADC were screened and 79 eligible patients using at least 2 types of ADCs with complete profiles were finally included. The baseline characteristics and treatment for all patients were summarized in Table 1. Patients ranged in age from 30 to 83 years, with 11(13.9%) were <40 years and 8 (10.1%) were >65 years. Among the 70 (88.6%) patients who underwent primary surgery, 75 patients received prior taxels-based chemotherapy (Table 1). HER2-positive disease was accounted for 64 (81.0%) patients (Table 1). According to the antibody and payloads of ADC1 and ADC2, 19, 53, 2, and 5 patients were classified in G1, G2, G3, and G4, respectively. Among 64 cases of HER2-positive MBC, 40, 13, and 11 of them received T-DM1, RC48, and T-Dxd as the first ADC, respectively (Supplementary Table S1). In 15 cases of HER2-low MBC, 6, 6, and 3 patients received SG, RC48, and T-Dxd as the first ADC, respectively (Supplementary Table S1). Nine patients were treated with 3 types of ADCs, the details are presented in Supplementary Table S2 and Supplementary Fig. S1.

**Table 1.** Patient characteristics.

Characteristics (*N* = 79)	Values
Age (years)	
Median (range)	51.7 (30-83)
Postmenopausal, *n* (%)	37 (46.8)
HER2 positive, *n* (%)	64 (81.0)
HER2 expression, *n* (%)	
IHC 1+	5 (6.3)
IHC 2+	23 (29.1)
FISH+	12 (15.2)
FISH−^a^	11 (13.9)
IHC 3+	51 (64.6)
HR positive, *n* (%)	35 (44.3)
Triple negative, *n* (%)	8(10.1)
Prior curative surgery, *n* (%)	70 (88.6)
Prior taxels-based therapy, *n* (%)	75 (94.9)
Prior anthracycline-based therapy, *n* (%)	47 (59.5)
Prior therapy for ADC1, *n* (%)	
Median (range)	3.29 (0-13)
Prior therapy for ADC2, *n* (%)	
Median (range)	5.48 (1-15)
Metastasis site, *n* (%)	
Non-regional lymph node metastasis	48 (60.8)
Lung	45 (57.0)
Bone	44 (55.7)
Liver	34 (43.0)
Brain	32 (40.5)
Treatment pattern for ADC1 and ADC2, *n* (%)	
T-DM1→T-Dxd	27 (34.2)
RC48→T-Dxd	15 (19.0)
T-DM1→RC48	13 (16.5)
T-Dxd→RC48	8 (10.1)
RC48→T-DM1	6 (7.6)
SG→RC48	4 (5.1)
T-Dxd→T-DM1	3 (3.8)
SG→T-Dxd	2 (2.5)
RC48→SG	1 (1.3)
Group, *n* (%)	
G1(similar antibody and payload)	19 (24.1)
T-DM1→RC48	13 (16.5)
RC48→T-DM1	6 (7.6)
G2(similar antibody but different payload)	53 (67.1)
T-DM1→T-Dxd	27 (34.2)
RC48→T-Dxd	15 (19.0)
T-Dxd→RC48	8 (10.1)
T-Dxd→T-DM1	3 (3.8)
G3(different antibody but similar payload)	2 (2.5)
SG→T-Dxd	2 (2.5)
G4(different antibody and different payload)	5 (6.3)
SG→RC48	4 (5.1)
RC48→SG	1 (1.3)

^a^one patient showed a negative result of HER2 FISH but HER2 enrich of PAM50 subtype.

Abbreviations: IHC, immunohistochemistry; ADC, antibody-drug conjugate; T-DM1, trastuzumab emtansine; T-Dxd, trastuzumab deruxtecan; SG, sacituzumab govitecan; RC48, disitamab vedotin.

At the end of the follow-up, 14 (17.7%) patients were still on treatment. The ORR was 10.1%, and 16.5% for ADC1 and ADC2, respectively (Supplementary Table S3). To investigate the impact of payload or antibody alternation on tumor response of ADC2, we focused on the proportion of patients who rapidly progressed in the first image evaluation, which could reflect the cross-resistance of ADCs. The ORR of ADC2 in patients treated with similar payloads was 5.3%. And the ORR of ADC2 in those treated with different payloads was as high as 22.6% (*P* = .180). In patients with rapid progression, the ORR of ADC2 was 9.1% and 41.2% in patients treated with similar payload and different payload, respectively (*P* = .397). For patients with a durable response of more than 6 months on ADC1, switching to a different payload of ADC2 showed an ORR of 15.0% and the ORR in patients treated with similar payload was 0% (*P* = .545). In HER2-positive disease, the proportion of PD in the first image evaluation with ADC2 in G1 and G2 was 42.1% and 37.8%, respectively (*P* = .96). For HER2-low disease, the proportion of PD at the first image evaluation of ADC2 in G2, G3, and G4 was 37.5%, 50.0%, and 40.0%, respectively (*P* = .95). The comparison for ORR of ADC2 is shown in Supplementary Fig. S2A.

**Figure 1.**
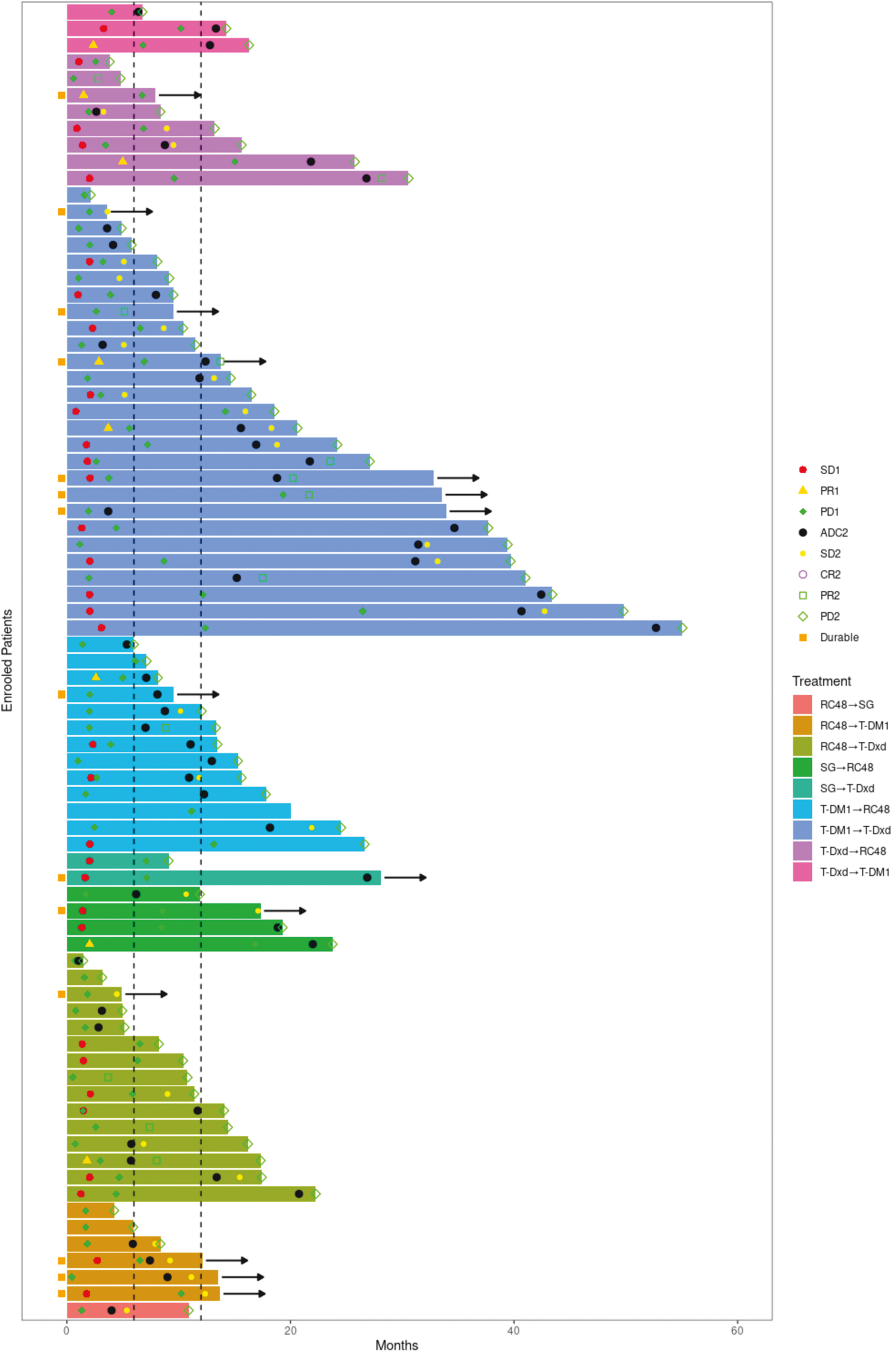
Swimmer plot of the patients receiving ADC1 and ADC2. PR1, SD1, PD1 stands for PR, SD, and PD for ADC1. ADC2 stands for the beginning of ADC2. PR2, CR2 SD2, PD2 stands for PR, CR, SD, and PD for ADC2.

**Figure 2.**
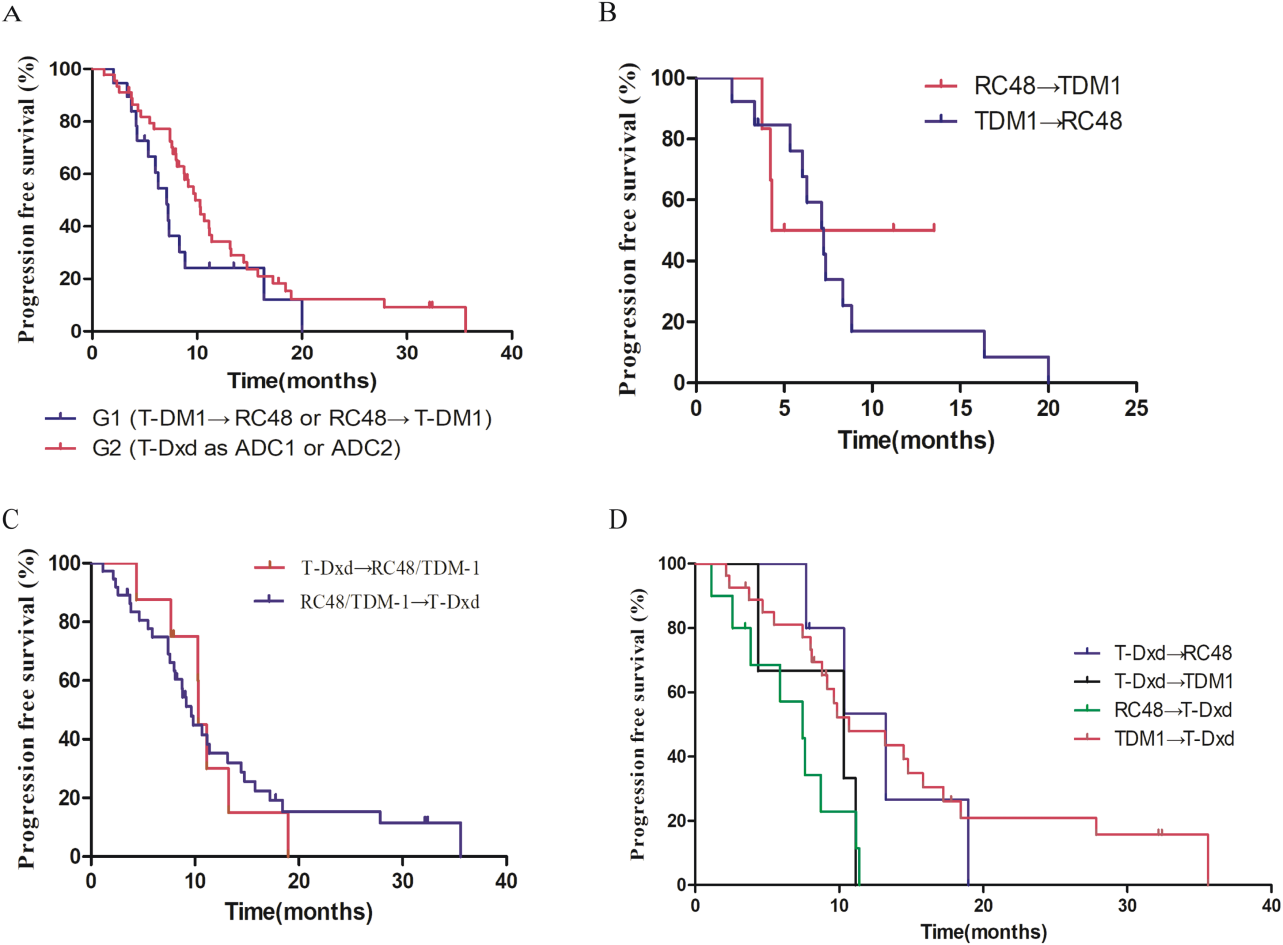
Progression-free survival (PFS) (A) for G1 and G2 in HER2-positive patients. PFS for RC48 and T-DM1 in different sequencing (B) and T-Dxd treated as ADC1 and that as ADC2 (C). The comparison of PFS for 4 combination strategy in G2 (D).

Median PFS_1_ and PFS_2_ for all patients were 3.23 and 3.93 months, respectively. The swimmer plot for all patients is shown in Figure 1. For HER2-positive cases, there was no significant difference in PFS was demonstrated between G1 and G2 (median PFS 7.13 months vs 9.83 months, hazard ratio [HR] = 1.72, 95% CI 0.86-3.41, *P* = .072, Fig. 2A). For patients in G1, similar PFS was seen in patients treated with RC48 and T-DM1 in different sequencing (RC48 followed by T-DM1 vs T-DM1 followed by RC48, median PFS 12.15 months vs 7.23 months, HR = 0.77, 95% CI 0.23-2.60, *P* = .921, Fig. 2B). In 45 patients in G2, there was no significant difference in PFS between T-Dxd treated as ADC1 and that treated as ADC2 (median PFS 10.3 months vs 9.63 months, HR = 1.08, 95% CI 0.46-2.54, *P* = .618, Fig. 2C). Among 4 combination strategies in G2, T-Dxd followed by RC48 achieved the longest PFS but no significant difference was seen between all groups (Fig. 2D).

**Figure 3.**
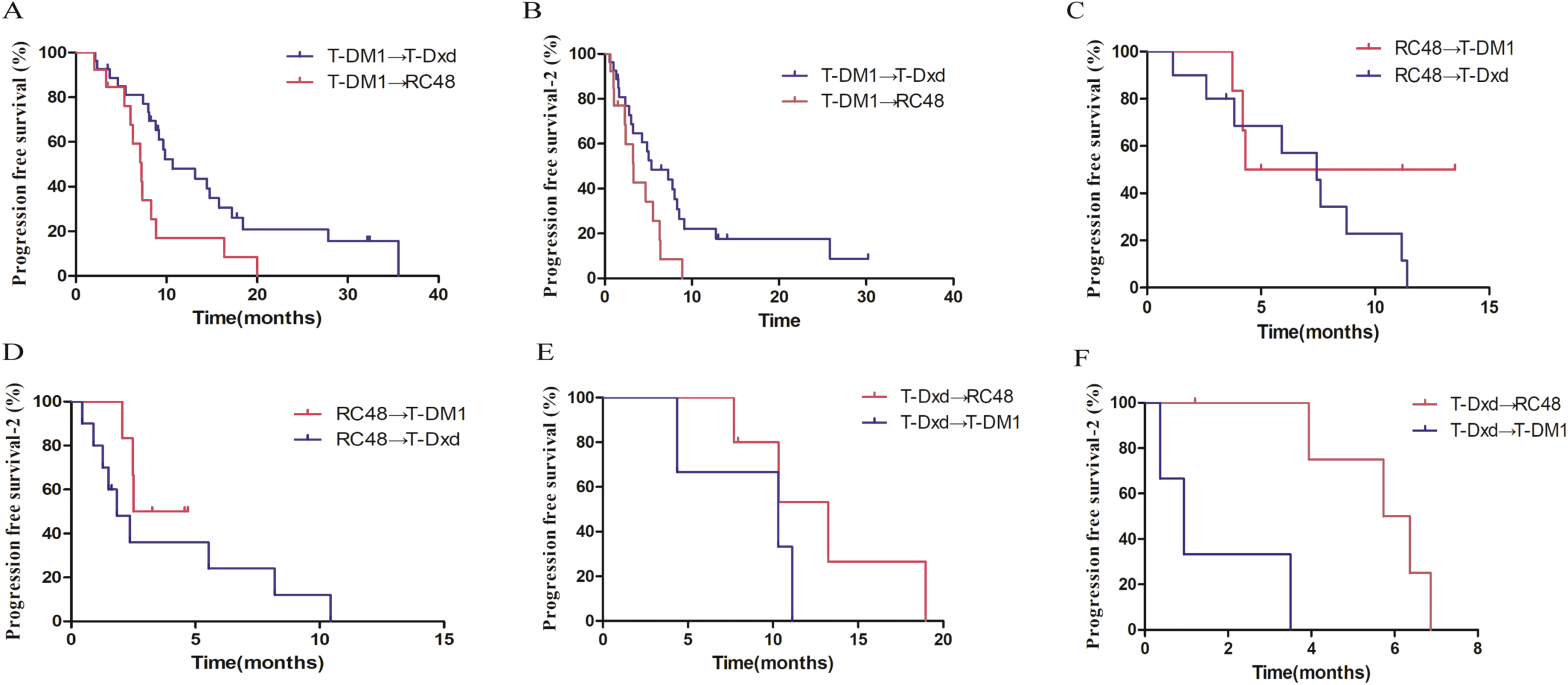
PFS and PFS_2_ of ADC2 for patients progressed after T-DM1 (A and B), RC48 (C and D), and T-Dxd (E and F).

To investigate the different patterns in HER2-positive MBC, we analyzed the PFS and PFS_2_ in patients who progressed after T-DM1, T-Dxd, and RC48, respectively. In 40 patients who progressed after T-DM1, both PFS and PFS_2_ of ADC2 were significantly longer in patients treated with T-Dxd compared with those treated with RC48 (median PFS 10.67 months vs 7.23 months, HR = 0.35, 95% CI 0.15-0.83, *P* = .017; median PFS_2_ 5.37 months vs 3.30 months, HR = 0.40, 95% CI 0.17-0.93, *P* = .034, Fig. 3A, 3B). In 16 patients who progressed after RC48, there was no significant difference for both PFS and PFS_2_ in patients treated with T-DM1 compared with those treated with T-Dxd (median PFS 8.90 months vs 7.43 months, HR = 0.53, 95% CI 0.16-1.71, *P* = .595; median PFS_2_ 6.47 months vs 1.83 months, HR = 0.49, 95% CI 0.13-1.84, *P* = .152, Fig. 3C, 3D). In 8 patients who progressed after T-Dxd, PFS_2_ was significantly longer in patients treated with RC48 compared with those treated with T-DM1, but similar PFS was demonstrated between 2 groups (median PFS_2_ 6.05 months vs 0.93 months, HR = 0.03, 95% CI 0.002-0.353, *P* = .0093; median PFS 13.2 months vs 10.3 months, HR = 0.29, 95% CI 0.04-1.88, *P* = .243 Fig. 3E, 3F).

For HER2-low patients, we analyzed the PFS for G2, G3, and G4, as well as patients treated with different patterns. The median PFS for G2, G3, and G4 was 6.35 months, 9.10 months, and 8.93 months, respectively (Supplementary Fig. S2B). Longer PFS was seen in patients treated with SG-containing regimens than in those treated with HER2 ADCs but no significant difference was seen (median PFS 9.10 months vs 6.35 months, HR = 0.50, 95% CI 0.15-1.60, *P* = .207, Supplementary Fig. S2C). As only one patient was treated with RC48 followed by SG, it was difficult to analyze the strategy for patients who progressed after HER2 ADC. In 6 patients who progressed after SG, no significant difference was seen between patients treated with RC48 and T-Dxd (Supplementary Fig. S2D).

We also analyzed the impact of tumor response to ADC1 on PFS_2_ for ADC2. Patients who progressed rapidly after ADC1 had similar PFS_2_ compared to those who did not progress in the first tumor evaluation (Supplementary Fig. S2E). We analyzed the PFS2 in intermediary and delayed treatment of ADC2. Among all included patients, no significant difference was shown in PFS2 for ADC2 in patients treated directly with ADC2 after progressing of ADC1 compared with those treated with other therapy and then received ADC2 (Supplementary Fig. S2F). In HER2-positive disease, median PFS2 was 4.87 months and 3.93 months in patients treated directly with ADC2 and those treated with other therapy and then received ADC2, respectively (Supplementary Fig. S3).

The median OS for all enrolled patients was 28.0 months, while the OS for HER2-positive patients and HER2-low patients was 28.0 months and 22.3 months, respectively. In patients who progressed from T-DM1, OS was significantly longer in patients treated with T-Dxd compared to those treated with RC48 (median OS 50.6 months vs 20.2 months, HR = 0.27, 95% CI 0.08-0.91, *P* = .034). While in patients who progressed after RC48, there was no significant difference for OS in patients treated with T-DM1 compared to those treated with T-Dxd. For HER2-low disease, superior OS was seen in patients treated with SG-containing strategy than that treated with HER2 ADCs but no significant difference was shown (median OS 31.9 months vs 22.0 months). Details of OS among different treatment strategies are shown in Supplementary Fig. S4.

**Figure 4.**
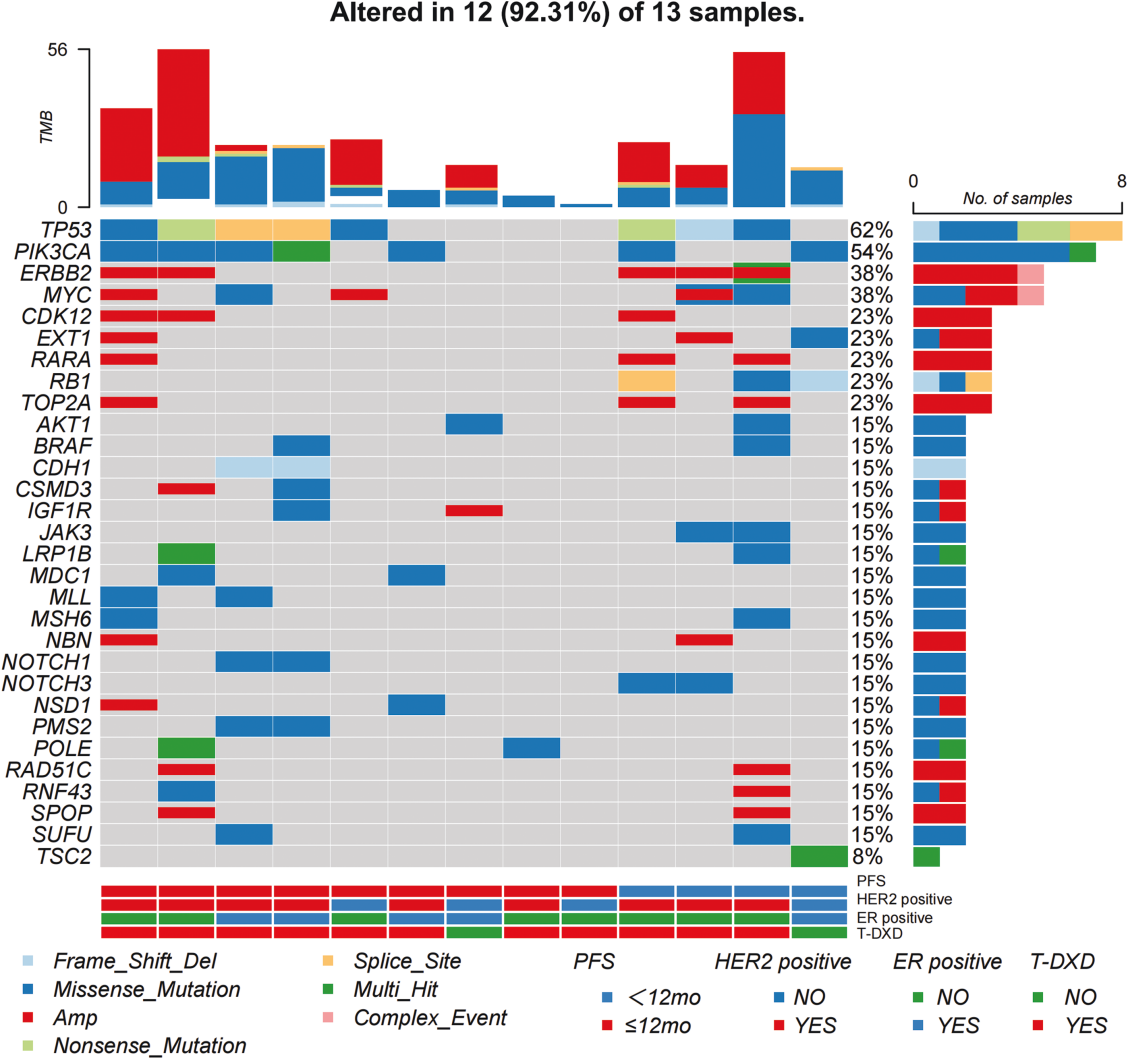
The overview of genomic alterations.

The landscape oncoplot of copy number alterations and nonsynonymous mutations for 13 samples with available NGS results of tumor sample is presented in Fig. 4. It was shown that 62% of the patients presented alternation of TP53 and 54% had PI3KCA mutation. Two cases of co-amplification of cyclin-dependent kinase 12 (CDK12) and ERBB2 were shown in 9 patients with PFS less than 12 months, compared to 1 in 4 patients with PFS longer than 12 months. In the subsequent profiling, we found that retinoblastoma1 (RB1) genes had different alteration trends between groups (*P* = .045), and the genomic alternation of RB1 was significantly associated with superior PFS.

## Discussion

In our study, we summarized patients with MBC treated with more than one type of ADCs and explored the optimal sequential use of ADC1 and ADC2. In HER2-positive MBC, RC48 showed inferior PFS_2_ and OS compared to T-Dxd after progression from T-DM1. However, T-Dxd showed similar PFS_2_ compared with T-DM1 after progression from RC48. Similar PFS was seen in patients treated with RC48 and T-DM1 in different sequencing, and in patients treated with T-Dxd and T-DM1 or RC48 in different sequencing. For HER2-low expression disease, it appeared that SG-containing regimens had longer PFS than those treated with 2 types of anti-HER2 ADCs. Furthermore, we analyzed the genomic landscape and indicated that RB1 mutation might be associated with superior PFS.

The use of ADCs has increased in recent years, and the diversification of antigenic targets as well as bioactive payloads is rapidly expanding the range of tumor indications. One challenge for ADCs is to determine the optimal sequencing for these agents.^1^ Maintained efficacy was seen in patients treated with second ADC with different payload in DESTINY-Breast 02 study and phase I trial of trastuzumab duocarmazine (SYD985), both T-Dxd and SYD985 showed significant activity in patients pretreated with T-DM1.^3,4^ In our study, we confirmed the benefit of PFS_2_ and OS of T-Dxd after treatment with T-DM1, which was consistent with the reported research. In the DESTINY-Breast 02 study, T-DM1 followed by T-Dxd achieved significantly superior PFS and OS compared to T-DM1 followed by capecitabine and trastuzumab or lapatinib (HR = 0.36), but the benefit for other HER2 ADCs after progression on T-DM1 was uncertain.^3^ In our study, T-DM1 followed by T-Dxd showed longer PFS2 compared to T-DM1 followed by RC48 (5.37 months vs 3.30 months, HR = 0.40), but the HR for T-Dxd was higher compared with that reported in DESTINY-Breast 02 study. This finding suggested that changing the payload might be an appropriate strategy for ADC sequencing. However, in patients pretreated with RC48, the benefit of T-Dxd compared with T-DM1 was unclear. T-Dxd is available since 2023 in China, but the incremental cost-effectiveness ratio was as high as 82 112 dollars per quality-adjusted life-year.^11^ T-Dxd is less cost-effective than T-DM1 for patients with HER2-positive MBC in China. In our study, most patients were treated with T-DM1 as ADC1 due to availability and financial toxicity. And we found that T-Dxd treated as ADC1 or as ADC2 presented similar PFS, suggesting that the sequencing of T-Dxd as second ADC could still be effective, especially for patients with higher financial toxicity.

The potential mechanism of resistance to ADCs is quite complex, including the antigen expression, ADC processing, and payload.^18,19^ HER2 heterogeneity is associated with resistance to T-DM1.^20^ Preclinical studies suggest that the bystander effect may overcome resistance to ADCs with noncleavable linkers, while cancer cells retain sensitivity to ADCs with cleavable linkers and cell-permeable payloads.^21^ In the DAISY study, resistance to T-DM1 was shown to be overcome by mechanisms including the bystander effect of T-Dxd.^22^ Among several combination strategy, T-Dxd followed by RC48 appeared to achieve the longest PFS in our study. RC48, a novel ADC with cleavable linker, showed promising antitumor activity in several types of HER2-positive tumors.^23^ We hypothesized that the bystander effect of T-Dxd and RC48 might overcome some of the drug resistance to achieve tumor response, but this strategy needs more evidence in the future. Based on the potential mechanism of resistance for ADCs, it was hypothesized that in patients resistant to tumor antigen, ADC1 followed by ADC2 with the same antigen but different payload would have a poor response, whereas, in patients with payload resistance, ADC1 followed by ADC2 with different payload would have a good response.^18^ In the A3 study, which focused the sequential use of ADCs in HER2-low expression MBC, it was showed that higher cases of cross-resistance were present when the second ADC contained the same antibody target as the first than when the second ADC targeted a different tumor antigen.^24^ In our study, the cases of cross-resistance as well as the PFS of ADC1 followed by ADC2 with a different payload is similar to that of ADC1 followed by ADC2 with similar payload in both HER2-positive and HER2-low expression disease, suggesting that the difference in the mechanism of resistance might have influence on sequential application. Further focus on how to determine the different mechanism of resistance is warranted.

The sequence of different ADCs with similar payloads for HER2-positive disease remained uncertain. Both T-DM1 and RC48 are effective in treating patients with HER2-positive BC who progress to taxanes, which, similar to DM1 and MMAE, act through microtubule disruption.^2,10^ Patritumab Deruxtecan, a HER3 ADC that carried the same payload as T-Dxd, might remain sensitive in patients who progress on T-Dxd with decreased HER2 expression.^25^ Thus, it is conceivable that the strategy using a similar chemotherapeutic agent may allow for continued antitumor activity. In our study, most patients progressed to taxanes. It was found that RC48 followed by T-DM1 seemed to achieve superior PFS than T-DM1 followed by RC48 (12.15 months vs 7.23 months), but the difference is not significant, indicating that the optimal strategy of continued microtubule disruption in different sequencing needs further research.

Understanding whether ADCs with similar payloads can be used in sequence may be particularly important for HR-positive MBC. T-DXd and SG, which carried a topoisomerase-I inhibitor as payload, seemed to have different survival outcomes on different sequencing. In the A3 study, which enrolled 34 patients with MBC, the PFS for T-Dxd followed by SG is superior to SG followed by T-Dxd.^24^ In the A3 study updated in SABCS 2023, the PFS1 for ADC1 was significantly longer than PFS2 for ADC2 (median TTP 5.36 months vs 2.56 months) in 68 HER2-low patients in USA, which suggesting the earlier treatment for ADCs.^13^ A similar conclusion was shown in Hupper’s report of 84 cases of HER2-low disease receiving SG and T-Dxd, the ORR was higher and real-world PFS was longer with ADC1 compared to ADC2, regardless of HR status and sequence.^12^ However, both the A3 study and Hupper’s report indicated that a few patients appeared to achieved more benefit from ADC2 than ADC1.^12^ Alternating antibodies but similar payloads did not seem to be the best strategy but may be appropriate in some special cases. In our study of HER2-positive and HER2-low patients, the median PFS1 for ADC1 and PFS2 for ADC2 were similar at 3.23 and 3.93 months, respectively. The difference in benefit for ADC1 and ADC2 between the A3 study, Huppert’s report, and our study may be due to the heterogeneous population. Our real-world study mainly included HER2-positive disease and most of them received T-DM1 as ADC1 due to drug availability and reimbursement, which was quite different from the A3 study. In our study, only 2 HER2-low patients received SG and T-Dxd, making it difficult to explore the sequential use for these agents because SG and T-Dxd were on the market in China since 2023. Some heavily treated HER2-low patients received RC48 instead. After progression from SG, RC48 may be an effective treatment, with similar PFS compared to T-Dxd in our research. More evidence is needed from larger sample size studies of SG and T-Dxd in HR-positive MBC is needed. Thus, more evidence is needed on the sequential use of ADCs for HER2-low disease in China. Poumeaud’s study reported 179 cases of MBC receiving T-Dxd followed by SG or SG followed by T-Dxd, with an mPFS2 of 2.7 months.^14^ The intermediate and delay treatment achieved 2.6 months and 3.1 months, respectively.^14^ In our study, the PFS2 for the intermediate line and no intermediate line was similar, which was consistent with the result in Poumeaud’s study reported at SABCS 2023.^14^ It was indicated that both intermediate and delayed treatment were suitable for patients, and the strategy for sequential use might depend on the identification of different mechanisms of ADC resistance.

Some studies have focused on the analysis of whole exome sequencing analysis of patients receiving ADCs who develop resistance. Mutations in TOP1 and TACSTD2, which encode topoisomerase-1 and TROP-2 respectively, were found to be associated with acquired resistance in SG.^26^ CDK12 is a member of the cyclin-dependent kinase (CDK) family, and genomic alterations in CDK12 have been reported in breast cancer. CDK12 induced anti-HER2 therapy inhibitor, while CDK12 inhibition is an effective strategy to inhibit tumor growth.^27^ In patients receiving HER2 tyrosine inhibitors, CDK12 inhibition suppressed PI3K/AKT signal and showed prominently benefit outcome.^28^ However, another research using biomarker analysis found that CDK12 amplification was associated with better response in patients receiving KN026, a biospecific HER2 antibody.^29^ Interestingly, in our study, CDK12 amplification appeared to be associated with similar PFS in our research. Further research on the controversial role of co-amplification of CDK12 and ERBB2 was warranted.

Germline mutations of the RB1 predispose to inherited retinoblastomas but also to other malignancies, including breast cancer.^30^ The role of somatic alternation of RB1 in breast cancer is controversial. Some studies have demonstrated that RB1 mutations are associated with resistance to anthracyclines in MBC and acquired resistance to T-Dxd in lung cancer,^31,32^ while functional loss of RB1 was significantly associated with higher response to neo-adjuvant trastuzumab and chemotherapy treatment in the NeoALTTO trial.^33^ In our study, we found that RB1 alternation was significantly associated with longer PFS, especially in HER2-positive patients, which is consistent with the finding in the NeoALTTO trial. The investigation of RB1 is warranted in further studies.

The limitations of this study lie in its retrospective nature and the heterogeneity in baseline characteristics and treatment factors, which might lead to potential bias. In addition, only 13 out of the 79 patients underwent NGS, and more genomic information is needed in the future. The main strength of the present study was that it analyzed the efficacy of sequential usage of ADCs in advanced breast cancer, especially in heavily treated patients. Therefore, further prospective clinical trials are warranted to confirm the optimal sequencing in a larger sample size.

## Supplementary Material

oyae055_suppl_Supplementary_Tables

oyae055_suppl_Supplementary_Figures

## Data Availability

The datasets generated during the current study are available from the corresponding author upon reasonable request.
