# Supplementary material for: Optimal Sequential Strategies for Antibody-Drug Conjugate in Metastatic Breast Cancer: Evaluating Efficacy and Cross-Resistance
Source: Oncologist. 2024 Apr 4;29(8):e957–66. doi: 10.1093/oncolo/oyae055 (PMC11299950; doi:10.1093/oncolo/oyae055)
Supplement: oyae055_suppl_Supplementary_Tables [file oyae055_suppl_supplementary_tables.pdf]

Supplementary Table S1. Summary of treatment for patients with HER-2 positive and HER-2 low disease.

Characteristics	HER-2 low (N=15)	HER-2 positive (N=64)	Overall (N=79)
**ADC1**			
T-Dxd	3 (20.0%)	11 (17.2%)	14 (17.7%)
RC48	6 (40.0%)	13 (20.3%)	19 (24.1%)
SG	6 (40.0%)	0 (0%)	6 (7.6%)
T-DM1	0 (0%)	40 (62.5%)	40 (50.6%)
**ADC2**			
T-Dxd	7 (46.7%)	34 (53.1%)	41 (51.9%)
RC48	7 (46.7%)	20 (31.3%)	27 (34.2%)
SG	1 (6.7%)	0 (0%)	1 (1.3%)
T-DM1	0 (0%)	10 (15.6%)	10 (12.7%)
**Group and Treatment**			
G1	0 (0%)	19 (29.7%)	19 (24.1%)
RC48→T-DM1	0 (0%)	6 (9.4%)	6 (7.6%)
T-DM1→RC48	0 (0%)	13 (20.3%)	13 (16.5%)
G2	8 (53.3%)	45 (70.3%)	53 (67.1%)
T-DM1→T-Dxd	0 (0%)	27 (42.2%)	27 (34.2%)
RC48→T-Dxd	5 (33.3%)	10 (15.6%)	15 (19.0%)
T-Dxd→RC48	3 (20.0%)	5 (7.8%)	8 (10.1%)
T-Dxd→T-DM1	0 (0%)	3 (4.7%)	3 (3.8%)
G3	2 (13.3%)	0 (0%)	2 (2.5%)
SG→T-Dxd	2 (13.3%)	0 (0%)	2 (2.5%)
G4	5 (33.3%)	0 (0%)	5 (6.3%)
RC48→SG	1 (6.7%)	0 (0%)	1 (1.3%)
SG→RC48	4 (26.7%)	0 (0%)	4 (5.1%)

ADC, antibody-drug conjugate; T-DM1, trastuzumab emtansine; T-Dxd, trastuzumab deruxtecan; SG, sacituzumab govitecan; RC48, disitamab vedotin.

Supplementary Table S2. Summary of treatment for nine patients receiving three types of ADCs.

	N	HER-2	Median PFS_1 for ADC1 (months)	Median PFS_2 for ADC2 (months)	Median PFS_3 for ADC3(months)
T-Dxd→RC48→TDM1	1	Positive	6.7	1.2	1.4
TDM1→RC48→T-Dxd	4	Positive	2.3	5.9	3.3
TDM1→T-Dxd→RC48	1	Positive	2.0	25.8	5.7
RC48→T-Dxd→TDM1	1	Positive	0.7	0.4	1.0
T-Dxd→RC48→T-Dxd	1	Positive	1.9	5.7	2.9
T-Dxd→RC48→SG	1	Low	9.6	3.8	2.3

Supplementary Table S3. Summary of best tumor response.

	**HER-2 low (N=15)**	**HER-2 positive (N=64)**	**Overall (N=79)**
**ADC1**			
ORR	2 (13.3%)	6 (9.4%)	8 (10.1%)
DCR	9 (60.0%)	32(50.0%)	41(51.9%)
PFS_1 (months)	3.00(1.67-8.57)	3.23(2.03-5.60)	3.23(2.47-5.00)
**ADC2**			
ORR	4 (26.7%)	9(14.1%)	13(16.5%)
DCR	8 (53.3%)	35(54.7%)	43(54.4%)
PFS_2 (months)	3.00(1.97-NA)	4.27(3.03-6.3)	3.93(3.00-5.7)
