# Supplementary material for: Optimal Sequential Strategies for Antibody-Drug Conjugate in Metastatic Breast Cancer: Evaluating Efficacy and Cross-Resistance
Source: Oncologist. 2024 Apr 4;29(8):e957–66. doi: 10.1093/oncolo/oyae055 (PMC11299950; doi:10.1093/oncolo/oyae055)
Supplement: oyae055_suppl_Supplementary_Figures [file oyae055_suppl_supplementary_figures.zip › Figures S1-S4/Supplementary Figures Legends.docx]

**supplementary FIGURE LEGENDS**

**Figure S1.** Swimmer plot of 9 patients were treated with three types of ADCs.

**Figure S2**. Objective response rate of ADC2 in different settings (A). Progression-free survival (PFS) of HER-2 low patients (B). The PFS in patients treated with SG containing regimens and that treated with HER-2 ADCs (C). The PFS for patients progressed after SG (D). The comparison of PFS_2_ in patients with or without rapidly progression after ADC1(E). The comparison of PFS_2_ in patients directly treated with ADC2 after progressed of ADC1 and those treated with other therapy then received ADC2 (F).

**Figure S3**. The comparison of PFS_2_ in patients directly treated with ADC2 after progressed of ADC1 and those treated with other therapy then received ADC2 in HEE-2 positive patients.

**Figure S4**. The overall survival (OS) for HER-2 positive and HER-2 low patients (A). The OS in patients progressed from T-DM1(B), RC48 (C). OS in HER-2 low patients (D).
