# Supplementary figures and images for: Optimal Sequential Strategies for Antibody-Drug Conjugate in Metastatic Breast Cancer: Evaluating Efficacy and Cross-Resistance
Source: Oncologist. 2024 Apr 4;29(8):e957–66. doi: 10.1093/oncolo/oyae055 (PMC11299950; doi:10.1093/oncolo/oyae055)

Figure S1

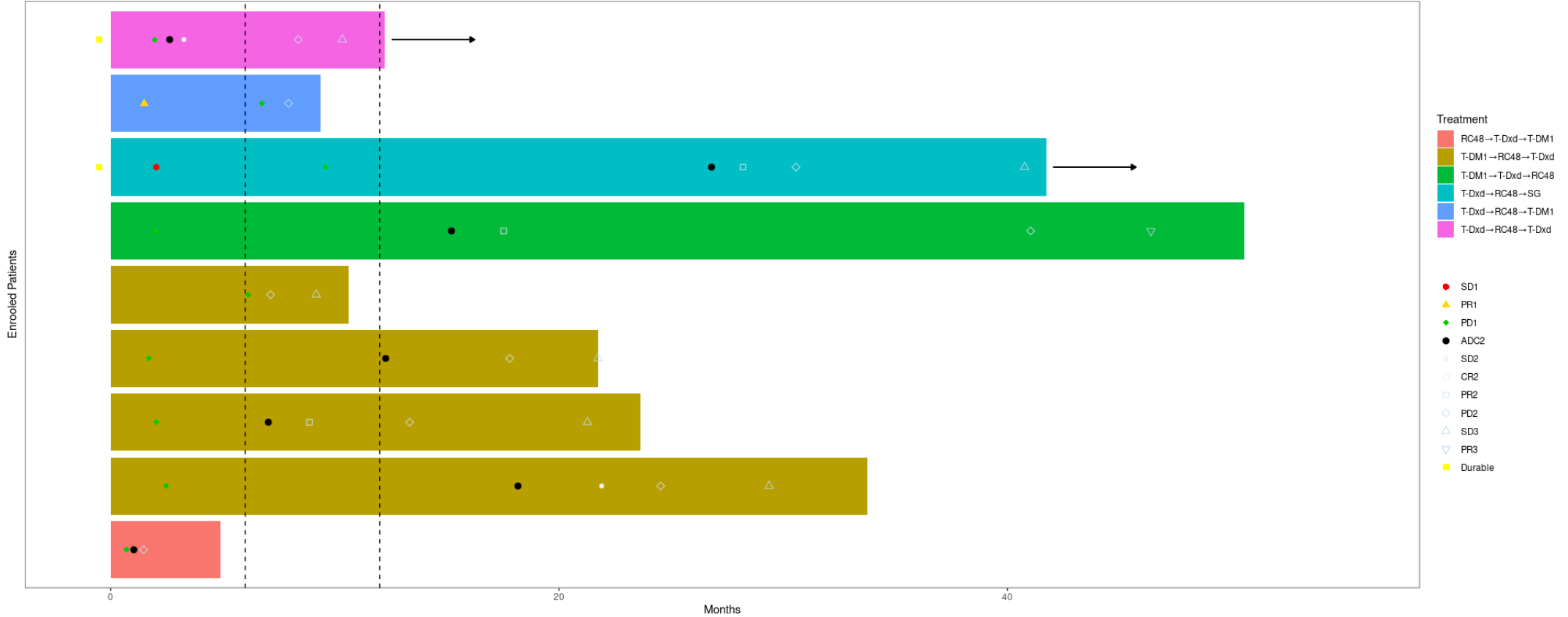

Supplement: oyae055_suppl_Supplementary_Figures [file oyae055_suppl_supplementary_figures.zip › Figures S1-S4/Figure S1.pdf]

A

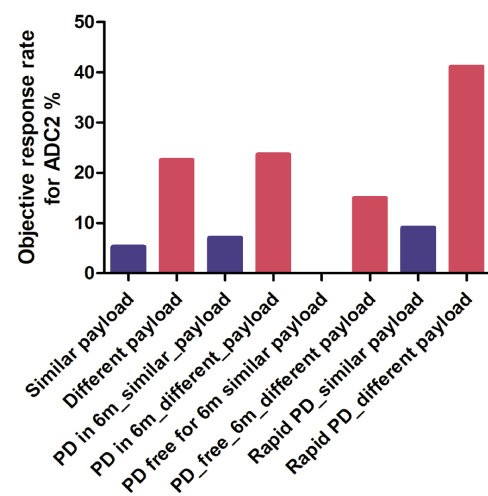

B

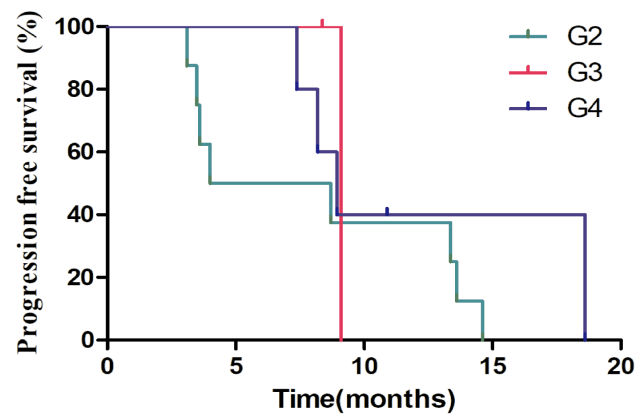

C

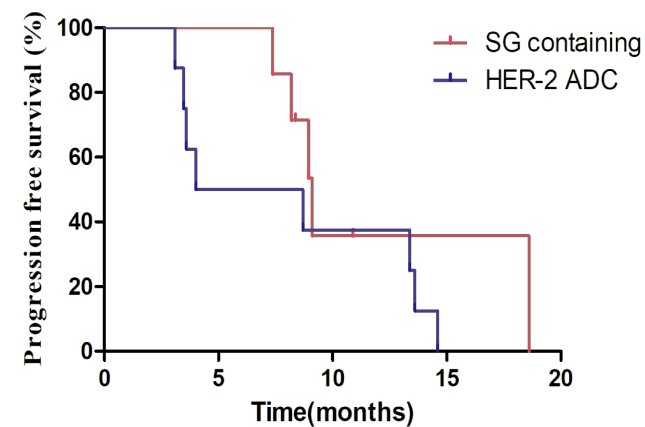

D

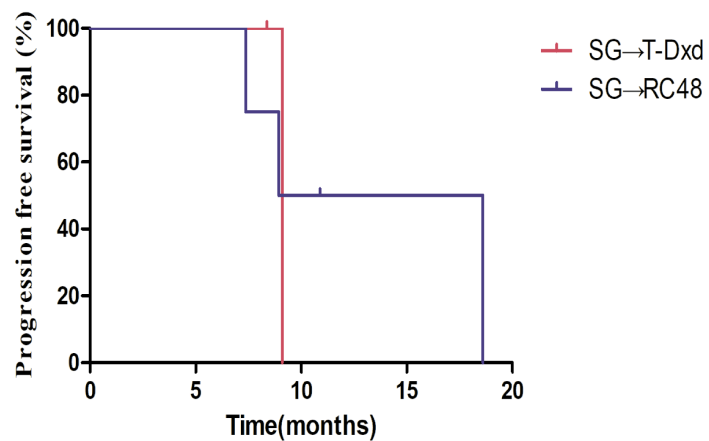

E

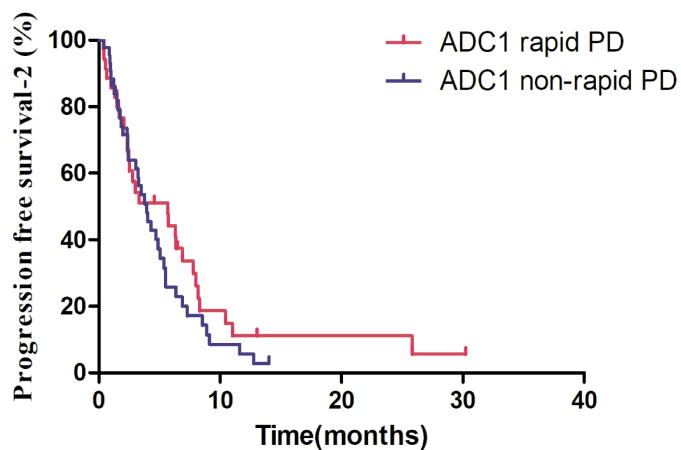

F

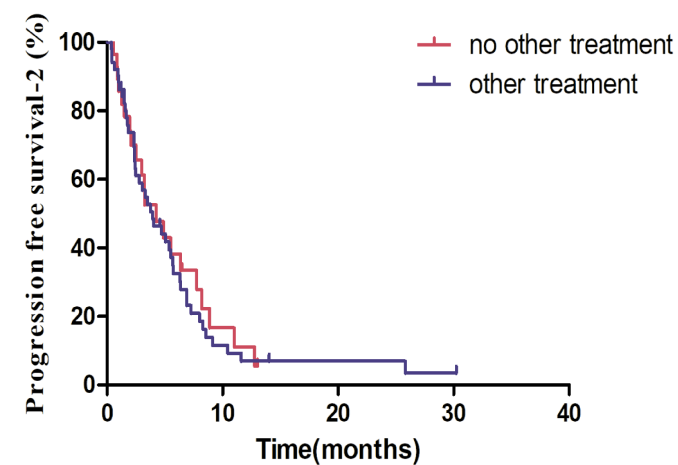

Supplement: oyae055_suppl_Supplementary_Figures [file oyae055_suppl_supplementary_figures.zip › Figures S1-S4/Figure S2_20231104.pdf]

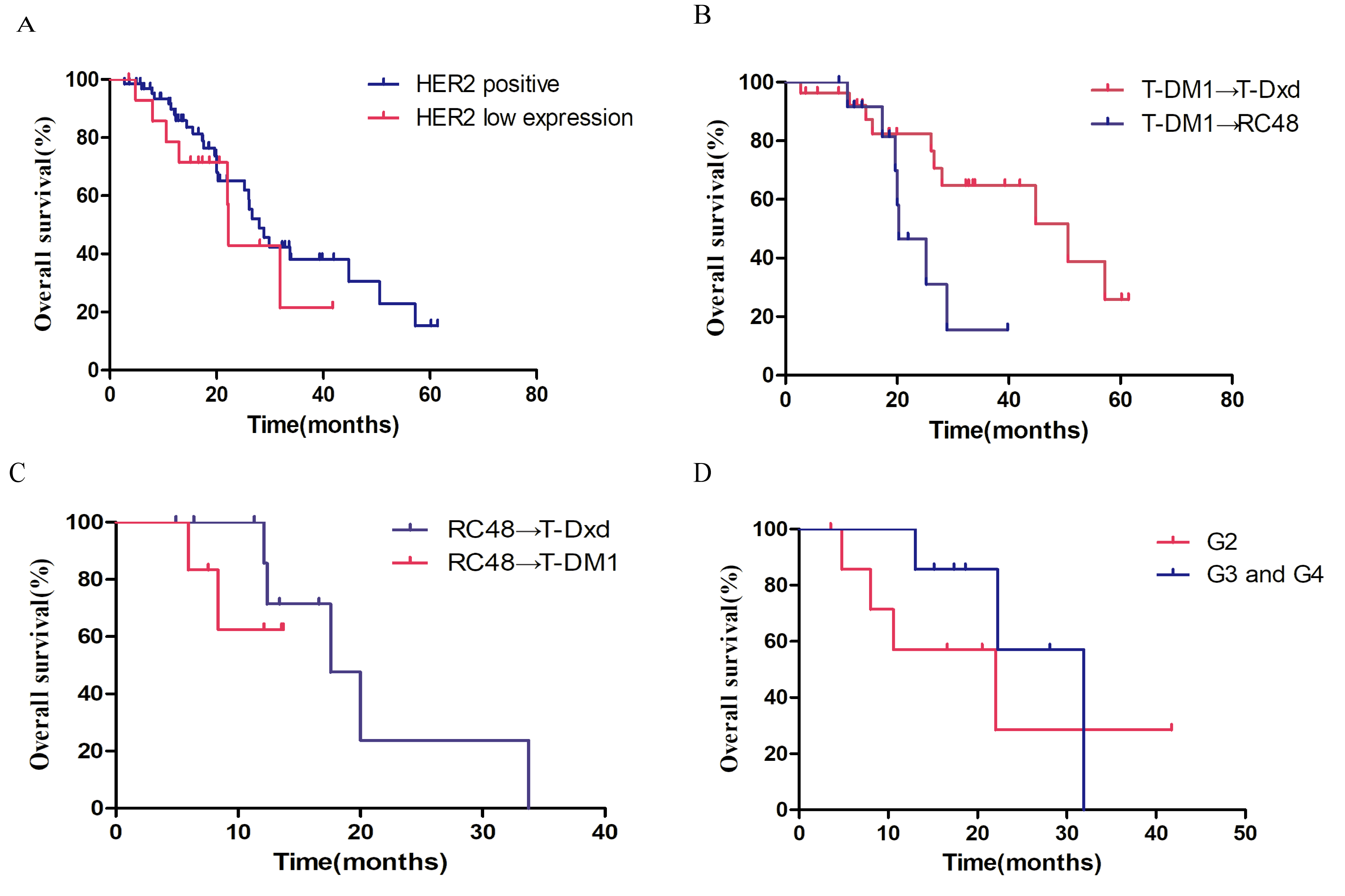

Supplement: oyae055_suppl_Supplementary_Figures [file oyae055_suppl_supplementary_figures.zip › Figures S1-S4/Figure S4_os.tif]
